# Egyptian protocol for living donor liver transplantation (LDLT) during SARS-CoV-2 pandemic

**DOI:** 10.1186/s43066-020-00074-4

**Published:** 2021-02-09

**Authors:** Hany Said Abd Elbaset, Ahmad Mohammad Sultan, Iman Fawzy Montasser, Hossam E. M. Soliman, Mohamed Elayashy, Nahed A. Makhlouf, Scientific Committee of Ministry of Health (MOH) National Project of Waiting Lists, Egypt

**Affiliations:** 1 HPB Surgery and Liver Transplantation, Ain Shams University, Cairo, Egypt; 2 GI Surgery, Liver Transplantation Unit, Gastrointestinal Surgical Center, Mansoura University, Mansoura, Egypt; 3 Tropical Medicine and Gastroenterology, Ain Shams University, Cairo, Egypt; 4 Professor of HPB Surgery and Liver Transplantation, National Liver Institute, Menoufia University, Menuofia, Egypt; 5 Anesthesia, Intensive Care and Pain Management, Cairo University, Cairo, Egypt; 6 Tropical Medicine and Gastroenterology, Medical Team of Liver Transplantation at Al-Rajhi Liver Hospital, Faculty of Medicine, Assiut University, Assiut, Egypt

**Keywords:** SARS-CoV-2, LDLT, Egyptian national protocol, Waiting list project

## Abstract

**Background:**

The current SARS-CoV-2 pandemic may negatively impact the care of liver transplant candidates and recipients.

**Main body of the abstract:**

Accordingly, each country must have its national guidelines based on the current situation and according to available tools. Liver Transplantation Scientific Committee of Waiting List Project in Egypt was established in 13 April 2020. One of the major objectives of this Scientific Committee is the preparation of national protocol for Transplant Centers in Egypt to deal with living donor liver transplantation (LDLT) during SARS-CoV-2 pandemic.

**Conclusions:**

The protocol highlights basic hospital requirements for LDLT during SARS-CoV-2 pandemic, the patient selection from the waiting list, management of patients on the waiting list, and post-transplant management.

**Supplementary Information:**

The online version contains supplementary material available at 10.1186/s43066-020-00074-4.

## Background

COVID-19 is the disease caused by an emerging coronavirus named severe acute respiratory syndrome coronavirus-2 (SARS-CoV-2) that was initially detected in Wuhan City, Hubei Province of China in December 2019. This virus spread across China and then spread worldwide and finally has been declared a pandemic [1, 2].

SARS-CoV-2 infection is acquired from anyone who is shedding the virus. Person-to-person transmission was detected through close exposure (< 6 feet) to an infected person with COVID-19, mainly via respiratory droplets produced when the infected case coughs or sneezes. The transmission frequently occurred from symptomatic persons with COVID-19 through droplet spread. Less frequently infection acquired from asymptomatic person has occurred, and the transmission is presumed to be possible from close contact with contaminated objects [1].

Older patients and those with pre-existing medical health problems are at risk of having severe course of disease. It is unclear to what degree chronic liver diseases should be considered as risk factors, due to insufficient clinical studies [3]. However, patients with end-stage liver disease are at increased risk of infection due to cirrhosis-associated immune dysfunction [4].

Between 1990 and August 2013, 3804 liver transplants were performed in Arab Countries, with Living Donor Liver Transplantation (LDLT) represented 80%. A lot of Egyptian patients were suffering from end-stage liver disease (ESLD), and necessitating liver transplantation (LT). More than 50% of transplanted cases in Arab countries were reported from Egypt. The regulations for LDLT were made by the Egyptian medical syndicate [5], whereas deceased donor (cadaveric) liver transplant (DDLT) has not yet been authorized. Between July 2018 and January 2020, 380 LDLT operations were performed in Egypt [Data were obtained from Ministry of Health (MOH)] registery database for National Project of Waiting Lists, Egypt).

Information on transplant recipients with COVID-19 are still few and based on published case reports or case series but accumulating experience is going on. However, based on previous data from other viruses including SARS, serious infection in the immunocompromised persons such as organ transplant recipients has happened. Mild affection has also been observed. The risk factors for severe infection have not been completely known. The American Society of Transplantation anticipated a greater viral load and shedding in transplant recipients that could result in more infectivity and spread to other persons [1]. The current pandemic requires unusual allocation of healthcare resources which may negatively impact the care of patients with chronic liver disease [3].

## Main text

### Rational

Each country with limited resources must have its national guidelines for LDLT during SARS-CoV-2 pandemic based on the current situation and according to the local resources and available tools.

### Aims of this protocol

1. Basic hospital requirements for LDLT during SARS-CoV-2 pandemic.
2. Patient selection from the waiting list.
3. Management of patients on the waiting list for LDLT.
4. Post-transplant management.

#### Hospital requirements for LDLT during SARS-CoV-2 pandemic

**Basic hospital staff, facilities, and equipment required to restart LDLT program during the pandemic:**

- Presence of sufficient staff for initial evaluation of cases based on symptoms, background, and exposure history (e.g., fever, respiratory symptoms, loss of taste or smell, contact with proven case of SARS-CoV-2 infection) by using rapid questionnaire survey.
- Nasopharyngeal swab for SARS-CoV-2 PCR laboratory facilities: Nasopharyngeal swab must be done for both recipient and donor 48 h before hospital admission and 24 h before the operation.
- High-resolution CT chest for both recipient and donor on hospital admission.
- Laboratory facilities and infection control team facilities for screening, early detection of suspected cases including hospital staff, potential donors, and recipients.
- Provide safe pathway for both recipient and donor including operative rooms, ICU, ward, necessary equipment such as ventilators, ultrasound, and duplex equipment.

**Medical staff precautions and protection:**

- Personal Protective Equipments (PPE) must be provided to all operative team, ICU staff, ward staff, radiology and laboratory staff i.e. (All medical persons and workers who are in contact with donor and recipient in perioperative care) (detailed infection prevention and control procedures during SARS-COV-2 pandemic, Additional file 1: Annex 1)
- Any medical staff with a history of contact with confirmed cases of COVID-19 without wearing full PPE must be temporary excluded from the team for home isolation for 14 days +/− nasopharyngeal swab if needed (i.e., if any suggestive symptoms appear like fever, cough) [6].

#### Patient selection from the waiting list

**Basic precautions needed for potential recipients for LDLT:**

Recipients of LDLT and donor candidates must have home isolation and personal distancing for a minimum of 10 days, counting back from the planned date of transplantation. Reporting any fever or respiratory symptoms to the transplant team by phone [7].

**Initial evaluation before admission:**

Evaluation is based on symptoms, background, and exposure history (e.g., fever, respiratory symptoms, loss of taste or smell, contact with proven case of SARS-COV-2 infection) and by the latest updated rapid survey questionnaire provided and updated by MOH.

**Nasopharyngeal swab for SARS-CoV-2 PCR:**

Nasopharyngeal swab must be done for both recipient and donor 48 h before hospital admission and 24 h before the operation [3, 7].

**High-resolution CT chest for both recipient and donor on hospital admission.**

As negative nasopharyngeal swab results do not completely exclude SARS-CoV-2 infection [8], so CT chest is mandatory during the pandemic.

**The transplant team must explain to potential recipients and their relatives the risk of SARS-CoV-2 infection aggravation under post-transplant immunosuppression** [7].

**Recipient selection,** Table 1.

**Table 1:** Recipient selection for LDLT during SARS-CoV-2 pandemic

	High priority risk group*	Moderate risk group
**Criteria**	● Fulminant liver failure ● MELD equal or greater than 20 ● Child-Pugh class C (score 10). ● Previous history of HRS. ● Previous history SBP. ● Benign (PVT) grade II. ● HCC within Milan unfit for bridge (Child-Pugh late B or C). ● HCC beyond Milan within UCSF with a good response of downstaging after 3 months.	● MELD 15—19 ● HCC within Milan fit for bridge therapy.
**Decision**	Proceed for transplantation with precautions	Complete donor preparation, close follow up on the waiting list

*MELD* model for end stage liver disease; *HRS* hepato-renal syndrome; *SBP* spontaneous bacterial peritonitis; *PVT* portal vein thrombosis; *HCC* hepatocellular carcinoma; *UCSF* University of California San Francisco;*  high priority  for liver transplantation  denotes mortality risk on the waiting list overweighs the risk of SARS-CoV-2 infection

*High priority for liver transplantation denotes mortality risk on the waiting list overweighs the risk of SARS-CoV-2 infection.

**Moderate risk**: Case by case discussion with Liver Transplantation Team, but the Scientific Committee of MOH national project of waiting lists does not recommend transplantation in the current situation.

**Transplant candidate with a history of confirmed COVID-19 disease:**

- Non-urgent transplantation should be avoided.
- If urgent transplantation is indicated:
- Nasopharyngeal/oropharyngeal PCR should be negative twice (at least 48 h apart).
- Complete resolution of clinical, laboratory, or radiological findings.
- Ideal disease-free interval is unknown but not less than 20 days from disease onset, and better to be more than 37 days [1, 9].

**Recommendations for the donors (pre-operative test and management)** [1, 3, 10]:

- Only the donor with low risk is accepted.

No direct contact with a positive or suspected patient.

No suspicious symptoms as follows:

- Fever (> 38 °C).
- Malaise or flu-like symptoms, +/− myalgias.
- New cough.
- Shortness of breath.
- Unexplained abdominal pain, nausea, and/or diarrhea.
- Loss of sense of taste and/or smell.

b) Recommendation for home isolation 14 days before hospital admission.
c) Complete blood count (CBC) shows no leucopenia and C-reactive protein (CRP) is negative
d) Radiological chest X-ray and high-resolution CT should be negative for any suspected lesion.
e) Nasopharyngeal/oropharyngeal PCR should be done twice in the week before transplantation with 48-72 h apart.
f) High-risk donors who have direct contact with positive or suspected patient with any suspicious symptom should be advised to postpone donation for 28 days after symptom resolution and have a negative PCR test.
g) Detailed informed SARS-CoV-2 infection risk consent should be taken from the donor [11]

#### Management of potential recipients on the waiting list for LDLT

- **a)** All potential recipients on the waiting list should be regularly followed up either by phone contact or transplant clinic visits whenever needed.
- **b)** Monthly update of Child-Pugh and MELD scores (weekly update if MELD> 25).
- **c)** Abdominal ultrasound/duplex and alpha-fetoprotein every 3 months.
- **d)** Triphasic CT portography if the results of ultrasound/duplex is suspicious.
- **e)** Weekly arrangement of waiting list by liver transplant team in each center according to Child-Pugh, MELD score, HCC characteristics of potential recipients.
- **f)** Mortality/dropout on the waiting list must be recorded including date, time on waiting list, and cause of death/dropout.
- **g)** We recommend that specialized centers for liver transplantation provide easily accessible contact information to facilitate immediate hepatology consultations to the local health care providers if needed.
- **h)** Include nasopharyngeal swab PCR testing for COVID-19 disease for patients with acute on chronic liver failure [3].
- **i)** Counseling of liver transplant candidates for vaccination against *Streptococcus pneumoniae* and influenza [3].
- **j)** Detailed informed Covid-19 disease risk consent for recipient before the operation [11]

#### Post-transplant management

**Immunosuppressant protocol:**

Maintain the usual protocol of immunosuppressants which is based on calcineurin inhibitors (CNIs), mycophenolate mofetil (MMF) and steroid, and at the usually recommended target levels.

**For a recipient with proven post-transplant Covid-19 disease:**

- Data from different liver transplant centers are scarce and there is no consensus on how to deal with immunosuppressant drugs in such situation. So far there have been no specific recommendations in terms of course or management of liver transplanted patients from major Societies. Some reports suggest decreasing immunosuppression for infected recipients, if no recent rejection episodes. Paradoxically, others suggest that a reactive immune response might be the cause for severe tissue damage and that immunosuppression might be protective from the postulated cytokine storm [12, 13].
- The national recommendations include reduction/hold of CNI dose, according to disease severity and radiological finding in CT chest. This must be done as case by case discussion in a multidisciplinary team with a senior chest consultant, transplant hepatology consultant, and ICU consultant in severe cases.
- **MMF/Azathioprine (AZA)** hold should be discussed on a case by case basis. It is essential to consider reducing azathioprine or mycophenolate dosages, especially in the setting of lymphopenia, fever, or worsening pneumonia attributed to COVID-19 [14].
- Patients who develop adult respiratory distress syndrome (ARDS), use steroid dose as recommended by Covid protocol prepared by MOH [15]. Methylprednisolone 1 mg/kg/day for 3-5 days then oral steroids in tapering dose over 4-6 weeks.
- Drug-drug interactions with immunosuppressant medications need to be evaluated and managed [15].
- We recommend regular telephone communication between the liver transplant team (or Central Transplant Committee) and health care physicians caring for liver transplant recipient with proven COVID-19 positive at isolation hospital.

**Post-transplant clinic and follow up visits:**

- For recipients who transplanted more than 3 months, the follow-up can be made through telemedicine with the transplant team according to scheduled visits as usual or exceptional telemedicine if any new developed symptoms or whenever needed. Hospital visit and/or admission whenever needed according to clinical judgment by the transplant physician in charge with the case [12, 16]. All recipients discharged from the hospital with educational booklet information that includes the alarm symptoms (e.g., fever, cough, vomiting, or diarrhea) and contact details for the transplant team members.
- Early postoperative period: less than 3 months from the operation, regular post-operative follow-up visits as per center protocol. Limit the number of patients who visit the transplant clinic, and limit the number of family members/friends who accompany patients in their visits. Special precautions on hand hygiene, wearing face mask throughout the visit, well-aerated rooms for waiting, keep distance 2 m between patients at waiting area, and decontamination of the waiting area should be practiced, etc. [12].
- Transplant recipients should be educated about the importance of performing frequent hand hygiene, cleaning frequently touched surfaces, avoidance of crowded public places, and applying social distancing, staying away from individuals who are ill [17].

**Vaccination:**

The usual vaccination of liver transplant recipients against *Streptococcus pneumoniae* and influenza is strongly recommended [3].

**Travel restriction for transplant recipients:**

It is essential to postpone all non-essential travel for transplant recipients. We also recommend that transplant patients’ immediate household contacts should not travel to high-risk areas [1]. Recipients transplanted from rural areas are instructed to stay nearby to their transplant centers at least for the first 3 months.

## Conclusion

The preparation of a national protocol for Transplant Centers in Egypt to deal with LDLT during SARS-CoV-2 pandemic is essential. This protocol highlighted basic hospital requirements, the patient selection from the waiting list, management of patients on the waiting list, and post-transplant management during SARS-CoV-2 pandemic. Regular and frequent updates are essentially needed according to any emerging data.

## Supplementary Information

**Additional file 1: Annex 1.** Infection Prevention and Control Procedures in Liver Transplant Centers During SARS-CoV-2 Pandemic.

## Data Availability

Not applicable.

## Abbreviations

LDLT: Living donor liver transplantation
SARS-CoV-2: Severe acute respiratory syndrome coronavirus-2
COVID-19: Coronavirus disease 2019
ESLD: End-stage liver disease
DDLT: Deceased donor (cadaveric) liver transplant
PPE: Personal protective equipments
MOH: Ministry of health
MELD: Model for end-stage liver disease
HRS: Hepato-renal syndrome
SBP: Spontaneous bacterial peritonitis
PVT: Portal vein thrombosis
HCC: Hepatocellular carcinoma
UCSF: University of California San Francisco
CNIs: Calcineurin inhibitors
MMF: Mycophenolate Mofetil
AZA: Azathioprine
ARDS: Adult respiratory distress syndrome
