# Supplementary material for: Egyptian protocol for living donor liver transplantation (LDLT) during SARS-CoV-2 pandemic
Source: Egypt Liver J. 2021 Feb 9;11(1):14. doi: 10.1186/s43066-020-00074-4 (PMC7871952; doi:10.1186/s43066-020-00074-4)
Supplement: Supplementary file 1 — Additional file 1: Annex 1. Infection Prevention and Control Procedures in Liver Transplant Centers During SARS-CoV-2 Pandemic. [file 43066_2020_74_MOESM1_ESM.doc]

**Annex 1**

**Infection Prevention and Control Procedures in Liver Transplant Centers During SARS-COV-2 Pandemic**

**Our aim** is to apply the optimum procedures to protect donor, recipient and health care workers form SARS-COV-2 infection in the perioperative time.

**Assigned personnel**: hospital infection control team in communication with transplant unit directory.

**General measures:**

**Applying standard precautions for all patients:**

- Standard precautions include hand and respiratory hygiene, the use of appropriate personal protective equipment (PPE)
- According to a risk assessment, injection safety practices, safe waste management, proper linens, environmental cleaning, and sterilization of patient-care equipment.
- Ensure that the following respiratory hygiene measures are used:
  - all patients should cover the nose and mouth with a tissue or using their elbow when cough or during sneezing
  - provide a surgical mask to cases with suspected COVID-19 during their presence in the waiting or public areas or in cohorting rooms.
  - practice proper hand hygiene after touching respiratory secretions.
- HCWs should apply WHO’s My 5 Moments for Hand Hygiene practice before their touch to a patient, before doing clean or aseptic procedure, after contact to body fluid or secretion, and after contacting a patient or his surroundings.
  - Hand hygiene includes either sterilizing hands with hand rub that contain alcohol or washing with soap and water.
  - Alcohol-based hand rubs are preferred if hands are not visibly contaminated
  - Wash hands with soap and water when they are visibly soiled.
- Consistent use of PPE correctly helps to minimize the spread of organisms. PPE effectiveness depends mainly on regular supplies, continuous staff training, proper hand hygiene, and appropriate human behavior.
- It is essential that cleaning and disinfection practices are used correctly. Cleaning touched surfaces and using hospital level disinfectants (such as sodium hypochlorite) are sufficient. Safe routine procedures should be followed in dealing with medical devices and equipment, laundry, food service utensils, and medical waste.

**Protecting Healthcare Workers and Workforce Utilization:**

- Cancel all in-person meetings (even small meetings) and change to virtual meetings.
- Practice social distancing even in meetings, e.g., keep an empty chair between each person.
- It is essential to restrict the direct patient care by health care providers who are considered at high risk (age more than 65 years, underlying medical problems, immunocompromised).
- Reduce or even prevent the presence of non-essential team persons in the hospital (e.g., students, observers, research staff) to reduce exposure risk and prioritize PPE use.
- Assign specific persons who will deal with patients during perioperative time.
- Ensure adequate training of medical staff about infection control procedures and correct donning and doffing of PPE.
- Use the least number of personnel according the procedure required.
- Create a backup schedule for physicians and surgeons in the event of quarantine or illness.
- Consider checking temperatures of all providers and staff as they arrive to the office or clinic.
- Use the PPE according to the procedure either aerosol generating or not.
- Consider equipping patient rooms with telemedicine equipment (e.g., tablet) to enable remote consultation and monitoring.
- Keep on hand hygiene on a regular basis before and after dealing with patients.

**Protecting Donor and Recipient:**

**Preoperative recommendations:**

- Donor and recipient should be home isolated at least 10 days before hospital admission.
- During isolation time, any symptoms like fever, sore throat or coughing developed for either donor or recipient should be informed by phone to the medical team and transplant should be cancelled.
- During admission, patients should be assessed adequately for any symptoms or signs of infection.
- Prepare specific isolated rooms for patient admission.
- Admission rooms should be cleaned and decontaminated first with 1% sodium hypochlorite or phenolic disinfectants.
- Recommended to admit patients 4 days before the procedure.
- Patients should be directly admitted to their room without passing through the emergency department or outpatient clinic to minimize personal contact.
- Limit the number of team members who enter a patient’s room for patient examinations and encounters.
  - The same rule applies to inpatient consultations including other services for the care of patients with liver disease or transplant evaluation. Limit the number of persons who enter patient rooms to the minimum required for doing consultative care.
  - Consider doing virtual visits for updates not needing direct evaluation. This will minimize contact risks as well as unnecessary using of PPE, and preserving hospital supplies for essential demands.
- Limit the number of visitors who may see inpatients, ideally, no visitors should be permitted in patient rooms.
- Patients should wear a surgical mask most of time during hospital stay when dealing with health care staff.
- Avoid unnecessary labs and imaging as possible.
- Pathways should be decontaminated first and free from pedestrians during any patients transfer.
- Decrease the distance of transfer between patient room, operating theatre and ICU as possible, preferred to be in the same zone.

**Intraoperative recommendations:**

- Cleaning and decontamination of operating room, electronic devices, and all theater contents with 1% sodium hypochlorite or specific disinfectant according to hospital policy.
- The patient should wear surgical mask till the time of anesthesia, and avoid unnecessary disconnection of breathing circuits.
- Reduce the number of persons in operating theatre to the minimum as required.
- All persons in the operating theatre should wear PPE (gown, surgical mask, overhead and gloves) N95 and face shield during aerosol generating procedure (intubation, suctioning, extubation).
- All doors of operating room should be closed most of the time.
- Keep regular checking of humidity and temperature.
- After extubation, if no need for supplemental oxygen by face mask, patient should wear a surgical mask during transfer to ICU.

**Postoperative recommedations:**

- Patients should be admitted in isolated rooms.
- Cleaning and decontamination of rooms before admission.
- All medical teams should wear PPE in case of contact with patients.
- Use full PPE including N95 and face shield during periods of airway communication or nebulizer sessions.
- Decrease number of personnel in patient room to the minimum.
- Decrease number of consultants and medical staff visits as possible and use telemedicine consultations.
- Avoid any relative visits during all postoperative stay and if necessary, should be restricted on one healthy person during all stay with sticking to all protective measures and proper distances.
- Regular assessment for symptoms and signs of infection.
- Any attacks of fever, sore throat, cough, respiratory symptoms or changes in chest imaging indicates nasopharyngeal swap for COVID19 PCR testing.
- Every center should have a detailed protocol for how to deal with and where to isolate suspected cases.

**Patients developed confirmed COVID-19 disease:**

- Early transplanted patients need special type of care and should be treated in the same hospital in an isolated place and not transferred to other hospitals.
- Ministry of health authorities should be informed.
- All medical team should stick to full PPE.
- All patient contacts without protective measures should be screened,
- All surrounding areas should be decontaminated regularly.
- Plan of treatment should start immediately with special consideration to immunosuppression drugs.
- Safety of medical personnel is a priority, no need for any rush without full PPE during emergencies.

**References for Annex 1:**

- World Health Organization. Infection prevention and control during healthcare when COVID-19 is suspected. Interim guidance 19 March 2020.
- World Health Organization. Infection prevention and control during health care for probable or confirmed cases of Middle East respiratory syndrome coronavirus (MERS-CoV) infection: interim guidance (accessed 17 January 2020).
- World Health Organization. Infection prevention and control of epidemic- and pandemic-prone acute respiratory diseases in health care. (Accessed 17 January 2020).
- World Health Organization. Guidelines on core components of infection prevention and control programs at the national and acute health care facility level. Geneva: World Health Organization; 2016 (accessed 20 January 2020).
- Minimum requirements for infection prevention and control. Geneva: World Health Organization; 2019 (<https://www.who.int/infectionprevention/> publications/min-req-IPC manual/en/, accessed 20 January 2020).
- WHO guidelines on hand hygiene in health care: first global patient safety challenge –clean care is safer care. Geneva: World Health Organization; 2009 (https://apps.who.int/iris/handle/10665/44102, accessed 17 January 2020).
- How to put on and take off personal protective equipment (PPE). Geneva: World Health Organization; 2008 (<http://www.who.int/csr/resources/publications/putonta> keoffPPE/en/, accessed 17 January 2020).
- Rational use of PPE.
- CDC and ICAN. Best Practices for Environmental Cleaning in Healthcare Facilities in Resource Limited Settings. Atlanta, GA: US Department of Health and Human Services, CDC; Cape Town, South Africa: Infection Control Africa Network; 2019. (<https://www.cdc.gov/hai/prevent/resourcelimited/> environmental-cleaning.html and <http://www.icanetwork.co.za/icanguideline2019/>, accessed 20 January 2020).
- Decontamination and Reprocessing of Medical Devices for Health-care Facilities. Geneva: World Health Organization; 2016 (<https://www.who.int/infectionprevention/> publications/decontamination/en/, accessed 20 January 2020).
- AASLD. American Association for the Study of Liver Diseases. Clinical Insights for Hepatology and Liver Transplant Providers During the COVID-19 Pandemic. Released: April 7, 2020
- ECDC Technical Report. Infection prevention and control and preparedness for COVID-19 in healthcare settings. Second update – 31 March 2020.
